# Distinguishing the impact of distinct obstructive sleep apnea syndrome (OSAS) and obesity related factors on human monocyte subsets

**DOI:** 10.1038/s41598-023-49921-5

**Published:** 2024-01-03

**Authors:** Ralph Pries, Friederike Katharina Kosyna, Reinhard Depping, Kirstin Plötze-Martin, Christian Lange, Svenja Meyhöfer, Sebastian M. Meyhöfer, Jens U. Marquardt, Karl-Ludwig Bruchhage, Armin Steffen

**Affiliations:** 1 Department of Otorhinolaryngology, University Hospital of Schleswig-Holstein, Campus Lübeck, Ratzeburger Allee 160, 23538 Lübeck, Germany; 2 Institute of Physiology, Working Group Hypoxia, Center for Structural and Cell Biology in Medicine, University of Lübeck, Lübeck, Germany; 3 Department of Medicine 1, University Hospital of Schleswig-Holstein, Lübeck, Germany; 4 Institute for Endocrinology & Diabetes, University Hospital of Schleswig-Holstein, Lübeck, Germany; 5 German Center for Diabetes Research (DZD), Neuherberg, Germany

**Keywords:** Immunology, Endocrinology, Molecular medicine

## Abstract

Obstructive sleep apnea syndrome (OSAS) and obesity go hand in hand in the majority of patients and both are associated with a systemic inflammation, immune disturbance and comorbidities such as cardiovascular disease. However, the unambiguous impact of OSAS and obesity on the individual inflammatory microenvironment and the immunological consequences of human monocytes has not been distinguished yet. Therefore, aim of this study was to investigate the impact of OSAS and obesity related factors on the inflammatory microenvironment by performing flow cytometric whole blood measurements of CD14/CD16 monocyte subsets in normal weight OSAS patients, patients with obesity but without OSAS, and patients with OSAS and obesity, compared to healthy donors. Moreover, explicitly OSAS and obesity related plasma levels of inflammatory mediators adiponectin, leptin, lipocalin and metalloproteinase-9 were determined and the influence of different OSAS and obesity related factors on cytokine secretion and expression of different adhesion molecules by THP-1 monocytes was analysed. Our data revealed a significant redistribution of circulating classical and intermediate monocytes in all three patient cohorts, but differential effects in terms of monocytic adhesion molecules CD11a, CD11b, CD11c, CX3CR1, CD29, CD49d, and plasma cytokine levels. These data were reflected by differential effects of OSAS and obesity related factors leptin, TNFα and hypoxia on THP-1 cytokine secretion patterns and expression of adhesion molecules CD11b and CD49d. In summary, our data revealed differential effects of OSAS and obesity, which underlines the need for a customized therapeutic regimen with respect to the individual weighting of these overlapping diseases.

## Introduction

It is well known that there is a close link between obesity and obstructive sleep apnea syndrome (OSAS) and that both conditions are overlapping in the majority of patients. Pathophysiologically, both entities are accompanied with systemic inflammation, cardiovascular disease as well as immune disturbance^1–4^. Several studies have linked the development and worsening of OSAS related hypoxia to weight gain^5^. Vice versa, OSAS gives rise to reduced physical activity and metabolic dysfunction, thereby promoting obesity^6^, which leads to immune cell recruitment to metabolic tissues and production of inflammatory mediators^7,8^. In adipose tissue, increased abundances of pro-inflammatory immune cells such as M1 macrophages and CD8^+^ T-cells have been observed^9^. Similarly, OSAS related hypoxia leads to an alteration of different immune cells such as lymphocytes, NK cells and monocytes and increased levels of inflammatory cytokines and adhesion molecules^10–14^. We have recently shown that OSAS patients reveal a redistribution of monocyte subsets followed by an imbalanced PD-1/PD-L1 communication with CD4/CD8 T cells. Interestingly, this clearly correlates with the individual body mass index (BMI)^15^. Our recent data revealed significantly decreased abundances of circulating classical monocytes accompanied by increased percentages of intermediate and non-classical monocytes in people with severe obesity (BMI ˃ 35 kg/m^2^). Plasma leptin levels, obstructive sleep apnea syndrome and diabetes status of these patients were identified as crucial amplifying factors, which underlines the impact of these comorbidities on the systemic immunity^16^.

However, the unambiguous impact of OSAS and obesity on the inflammatory microenvironment and the immunological consequences of human monocytes has not been considered separately.

Therefore, we analyzed monocyte subset abundances and characteristics in normal weight OSAS patients (OSAS cohort), patients with obesity but without OSAS (obesity cohort), and patients with OSAS and obesity (OSAS/obesity cohort), compared to healthy controls. In addition, OSAS and obesity related plasma levels of adiponectin, leptin, lipocalin, and matrix metallopeptidase 9 (MMP-9) were correlated with monocyte subset measurements and clinical characteristics to explicitly distinguish these two conditions. Furthermore, using the human monocyte leukemia cell line THP-1 (Tohoku Hospital Pediatrics-1)^17^, we analyzed the impact of distinct OSAS and obesity related parameters like hypoxia, tumor necrosis factor α (TNFα) and leptin on the secretion patterns of relevant cytokines, chemokines and cellular adhesion molecules and chemokine receptors.

The study aimed to better understand the context of OSAS and obesity and its specific impact on the immunological balances of circulating monocytes and the inflammatory microenvironment.

## Results

### OSAS and obesity related monocyte subset alterations

**Figure 1:**
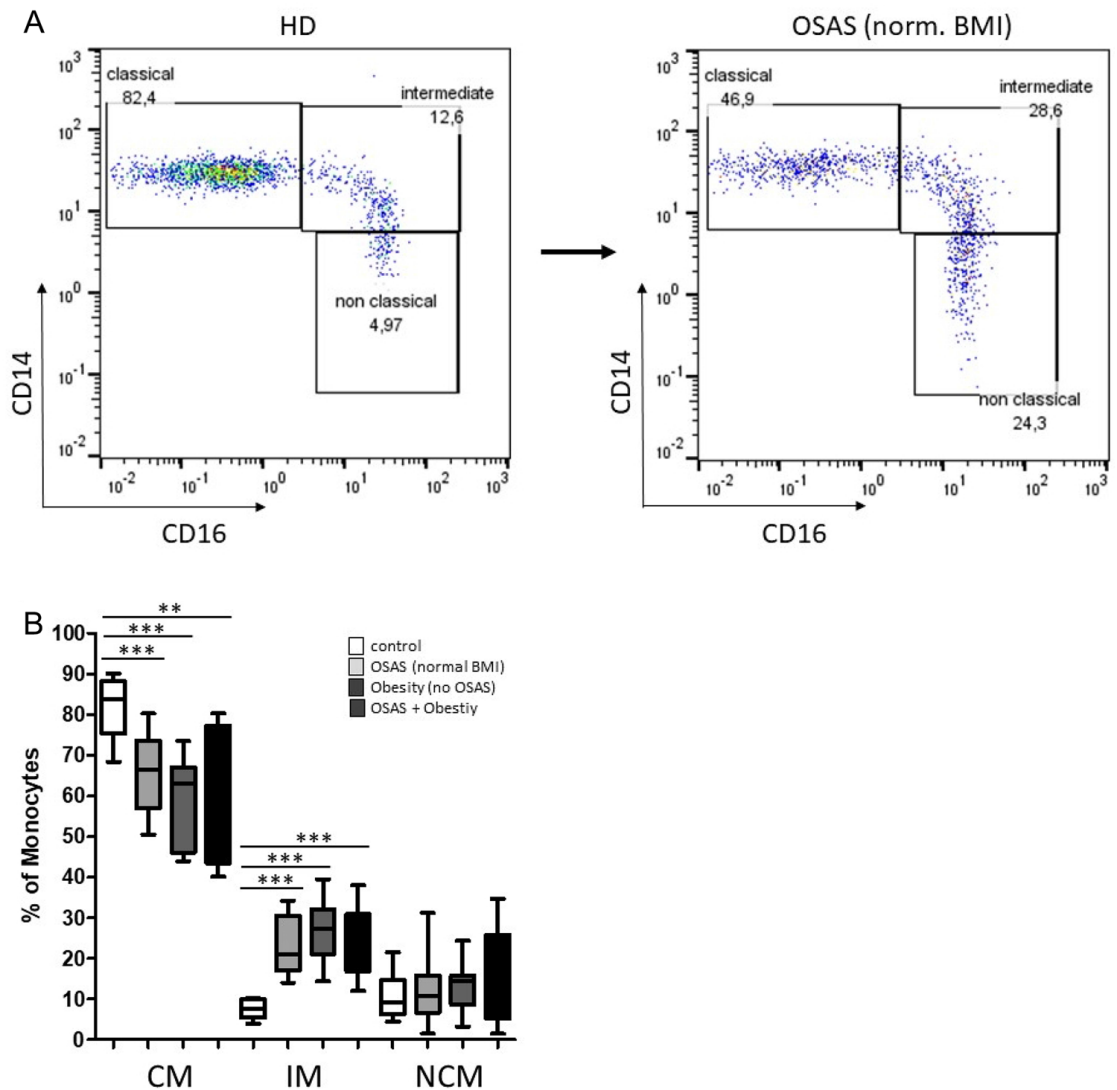
Flow cytometric analysis of CD14/CD16 characterized monocyte subsets. (**A**) Representative example gating scheme of flow cytometric analysis of peripheral monocyte subsets in a healthy donor (HD) and a normal weight OSAS patient. (**B**) Whole blood measurements revealed significantly decreased abundances of classical monocytes (CM) accompanied by significantly increased percentages of intermediate monocytes (IM) in obese patient without OSAS (n = 10), in OSAS patients with normal BMI (n = 10), and in patients with OSAS and obesity (n = 10), compared to healthy donors (n = 10). **: *p* < 0.01; ***: *p* < 0.001.

OSAS and obesity are accompanied by changed levels of different circulating cytokines, chemokines and adipokines, which are significantly involved in inflammatory processes and immune disturbance and the development of associated diseases. Whole blood measurements of CD14/CD16-characterized monocyte subsets were carried out as previously described using flow cytometry^15^. Therefore, monocytes were first roughly gated by their FSC/SSC characteristics and CD14/CD16 expression patterns. Next, neutrophil granulocytes, NK-cells and B-lymphocytes were excluded by means of HLA-DR and remaining monocytes were finally subgated into CD14^++^CD16^-^ (classical), CD14^++^CD16^+^ (intermediate) and CD14^dim+^CD16^+^ (non-classical) monocyte subsets (Fig. 1A).

We identified significantly decreased abundances of classical monocytes in normal weight OSAS patients (*p* = 0.0006), in patients with obesity but without OSAS (*p* =  < 0.0001), and in patients with OSAS and obesity (*p* = 0.0012), all accompanied by significantly increased percentages of the corresponding intermediate monocyte subset (*p* =  < 0.0001) (Fig. 1B).

**Figure 2:**
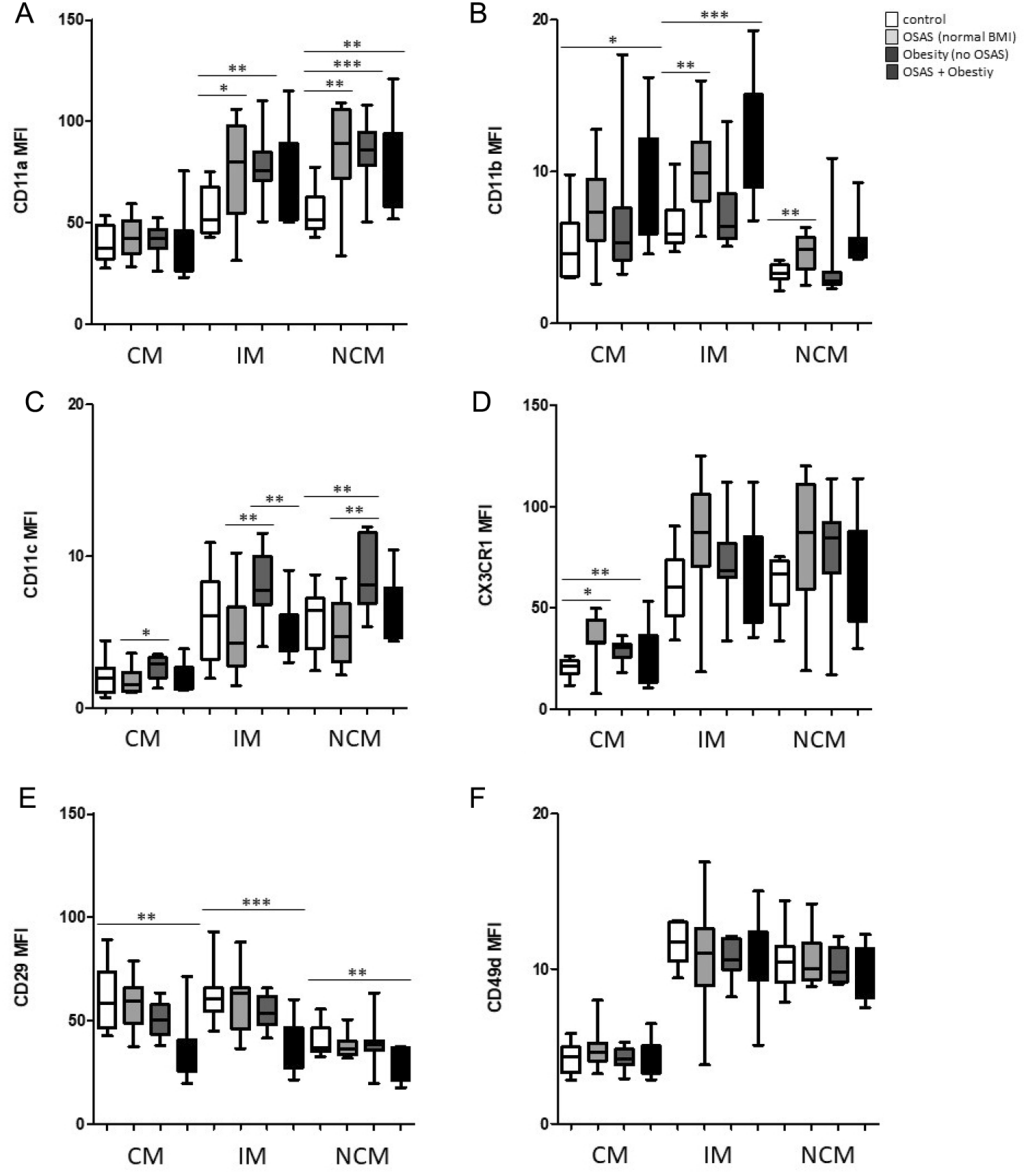
Flow cytometric analyses of monocytic adhesion molecules in normal weight OSAS patients (n = 10), patients with obesity without OSAS (n = 10), and patients with OSAS and obesity (n = 10), compared to healthy donors (n = 10). (**A**) The expression of CD11a was significantly elevated on intermediate monocytes (IM) from normal weight OSAS patients (n = 10), and patients with obesity without OSAS (n = 10). Percentages of non-classical monocytes (NCM) from all three patient cohorts were significantly increased compared to healthy donors (n = 10). (**B**) The expression of CD11b was significantly higher on classical monocytes and non-classical monocytes from patients with OSAS and obesity. Furthermore, CD11b expression was significantly higher on intermediate monocytes and non-classical monocytes from normal weight OSAS patients (n = 10) compared to healthy donors (n = 10). (**C**) The expression of CD11c was significantly higher on non-classical monocytes from in patients with obesity without OSAS (n = 10) compared to healthy donors (n = 10). CD11c was significantly higher on all three monocyte subsets in patients with obesity without OSAS (n = 10) compared to normal weight OSAS patients (n = 10). (**D**) CX3CR1 expression was significantly higher on classical monocytes (CM) in normal weight OSAS patients (n = 10) and patients with obesity without OSAS (n = 10) compared to healthy donors (n = 10). (**E**) Expression levels of CD29 were significantly decreased on all three monocyte subsets from patients with OSAS and obesity compared to healthy donors. (**F**) Expression levels of CD49d revealed no significant differences *: *p* < 0.05; **: *p* < 0.01; ***: *p* < 0.001.

Furthermore, expression of adhesion molecules and chemokine receptors CD11a (integrin-α L; LFA-1), CD11b (integrin-α M; Mac-1), CD11c (integrin-α X), CD29 (integrin β-1), CD49d (integrin β-4), and CX3CR1 (CX3CL1 receptor) were investigated. The expression levels of CD49d revealed no significant differences between the analyzed cohorts (Fig. 2). The expression of CD11a was significantly higher on intermediate monocytes from normal weight OSAS patients (*p* = 0.0423) as well as in the obesity cohort (*p* = 0.0028), and also on non-classical monocytes from both patient cohorts (*p* = 0.0056; *p* =  < 0.0001) as compared to healthy donors (Fig. 2A). The expression of CD11b was significantly higher on intermediate monocytes (*p* = 0.0031) and non-classical monocytes (*p* = 0.0066) from normal weight OSAS patients compared to healthy donors and revealed strong heterogeneous distributions among the analysed patient cohort (Fig. 2B). The expression of CD11c was significantly higher on non-classical monocytes in the obesity cohort (*p* = 0.0070) as compared to healthy donors (Fig. 2C).

Of note, the expression of CD11c was significantly higher on all three monocyte subsets in the obesity cohort (CM: *p* = 0.0187; IM:* p* = 0.0048; NCM: *p* = 0.0015) as compared to normal weight OSAS patients (Fig. 2C). The expression of chemokine receptor CX3CR1 was significantly higher on classical monocytes from normal weight OSAS patients (*p* = 0.0029) and patients with obesity (*p* = 0.0029) as compared to healthy donors (Fig. 2D). Interestingly, expression levels of CD29 were significantly decreased on all three monocyte subsets from patients with OSAS and obesity compared to healthy donors, but not in the OSAS or obesity cohort, suggesting an additive effect of both inflammatory conditions (Fig. 2E).

### OSAS and obesity related plasma adipokines

**Figure 3:**
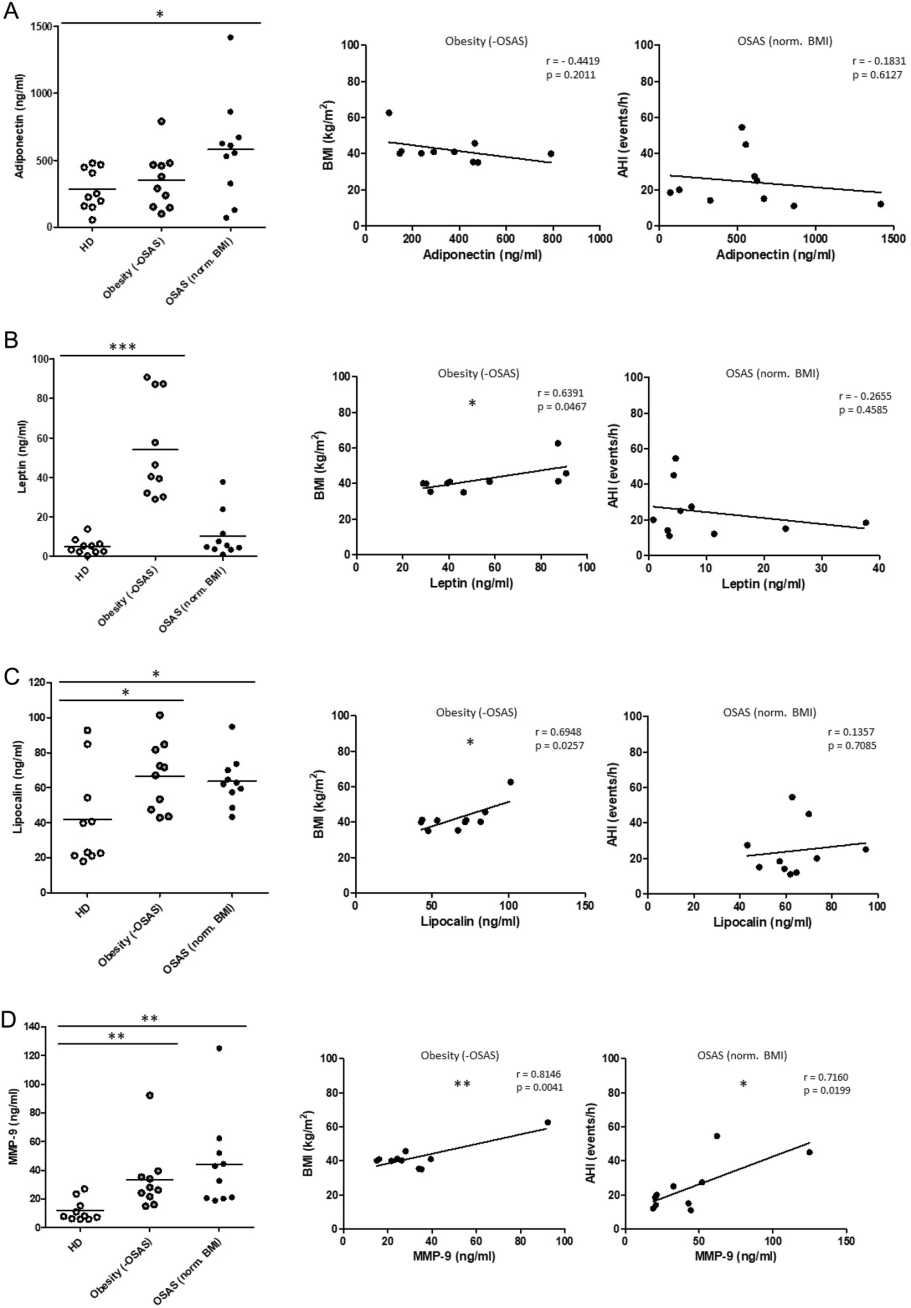
ELISA measurements of plasma adipokines and correlation BMI and AHI values. (**A**) Data revealed significantly increased plasma levels of adiponectin in normal weight OSAS patients (n = 10) compared to healthy donors (n = 10) but no significant correlations with BMI (kg/m^2^) and AHI (events/h) values. (**B**) Significantly increased plasma leptin levels were detected in patients with obesity without OSAS (n = 10) and revealed a significantly positive correlation with the BMI values of the analyzed patient cohort. (**C**) Plasma lipocalin levels were significantly higher in normal weight OSAS patients (n = 10) and in patients with obesity without OSAS (n = 10) and significantly correlated with the BMI values. (**D**) Plasma MMP-9 levels were significantly higher in both normal weight OSAS patients (n = 10) and in patients with obesity without OSAS (n = 10) compared to healthy donors (n = 10). There was a significant correlation between plasma MMP-9 levels and both the BMI and AHI values of the corresponding patient cohort. The Pearson correlation coefficient (r) and p values are given. *p* < 0.05 was considered as significant. *: *p* < 0.05; **: *p* < 0.01; ***: *p* < 0.001.

To further explicitly distinguish the specific impact of OSAS and obesity on systemic immunological balances, we analyzed plasma levels of adiponectin, leptin, lipocalin and metalloproteinase 9 (MMP-9) from normal weight OSAS patients and patients with obesity without OSAS using ELISA measurements. Data were further correlated with BMI and apnoe-hypopnea-index (AHI). Our data revealed heterogeneous but significantly increased plasma levels of adiponectin in normal weight OSAS patients (*p* = 0.0357) compared to healthy donors but no significant correlation with AHI values (Fig. 3A). Plasma leptin levels were significantly increased (*p* =  < 0.0001) and significantly correlated with the BMI in the obesity cohort. (Fig. 3B).

Plasma lipocalin levels were significantly elevated in normal weight OSAS patients (*p* = 0.0383) as well as in the obesity cohort (*p* = 0.0318) and significantly correlated with BMI (Fig. 3C). Plasma MMP-9 levels were significantly elevated in the OSAS cohort (*p* = 0.0065) as well as in the obesity cohort (*p* = 0.0099). Data revealed a significantly positive correlation between plasma MMP-9 and both the BMI and AHI values of the corresponding patient cohort (Fig. 3D).

**Figure 4:**
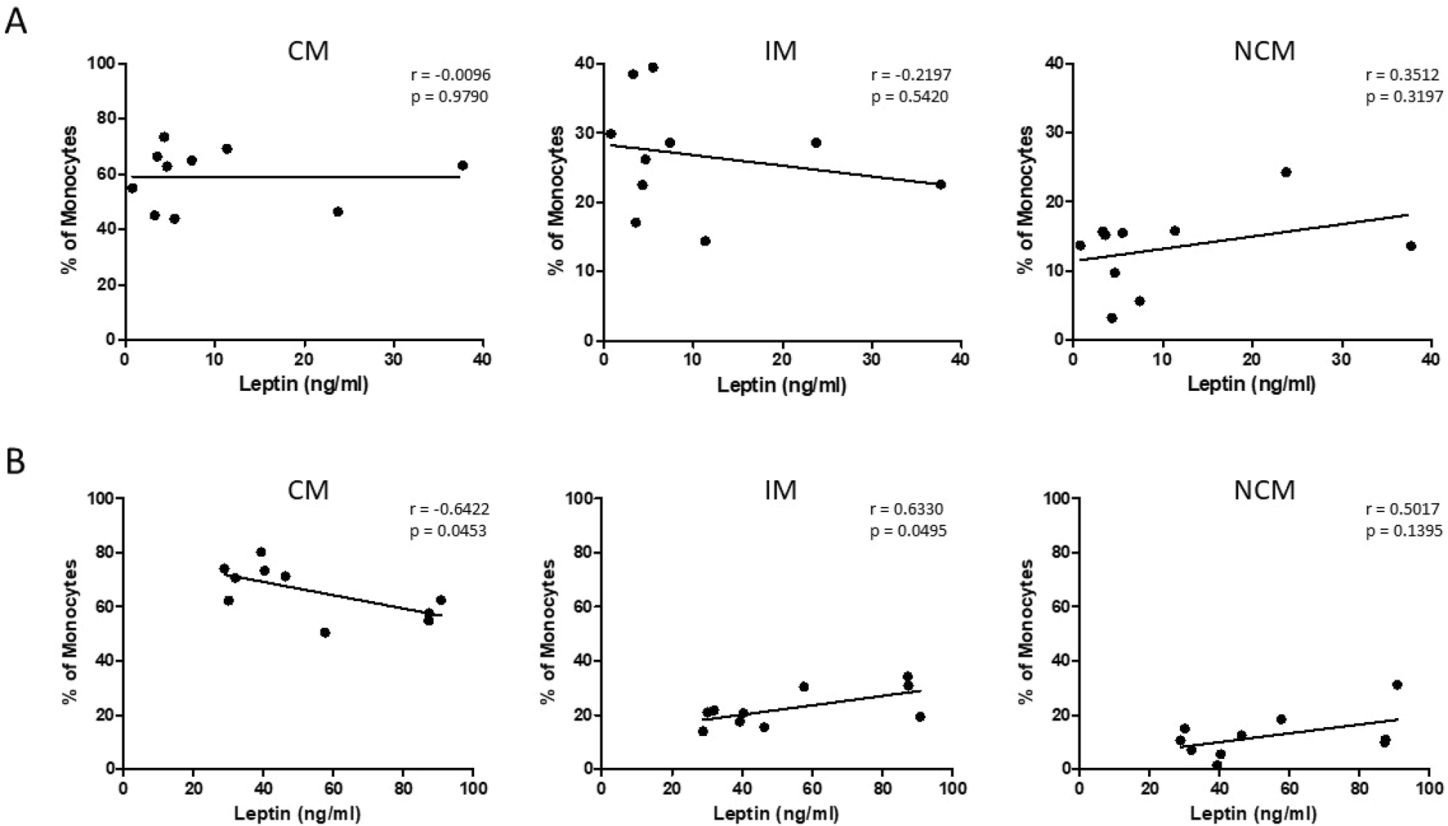
Correlation between monocyte subset abundances and plasma leptin levels. Correlation analysis between the percentages of classical monocytes (CM), intermediate monocytes (IM) and non-classical monocytes (NCM) and the plasma leptin values of (**A**) normal weight OSAS patients (n = 10) and (**B**) patients with obesity but without OSAS (n = 10) revealed a significant correlation with CM and IM solely from patients with obesity without OSAS, but not from normal weight OSAS patients. The Pearson correlation coefficient (r) and p values are given. *p* < 0.05 was considered as significant.

However, further correlation analyses revealed significant correlations between the measured plasma leptin levels and percentages of classical monocytes (CM; *p* = 0.0117), intermediate monocytes (IM;* p* = 0.0143), and non-classical monocytes (NCM;* p* = 0.0398) in the obesity cohort but not in the OSAS cohort (Fig. 4).

### Impact of OSAS and obesity related factors on THP-1 monocytes

We further analyzed the secretion patterns of 105 different cytokines and chemokines by THP-1 monocytes in response to 24 h incubation with OSAS and obesity related parameters hypoxia, tumor necrosis factor α (TNFα) and leptin using membrane based human cytokine arrays. Therefore, we used the THP-1 monocyte cell line as a reproducible model system and to exclude the complex individual parameters of primary cells.

**Figure 5:**
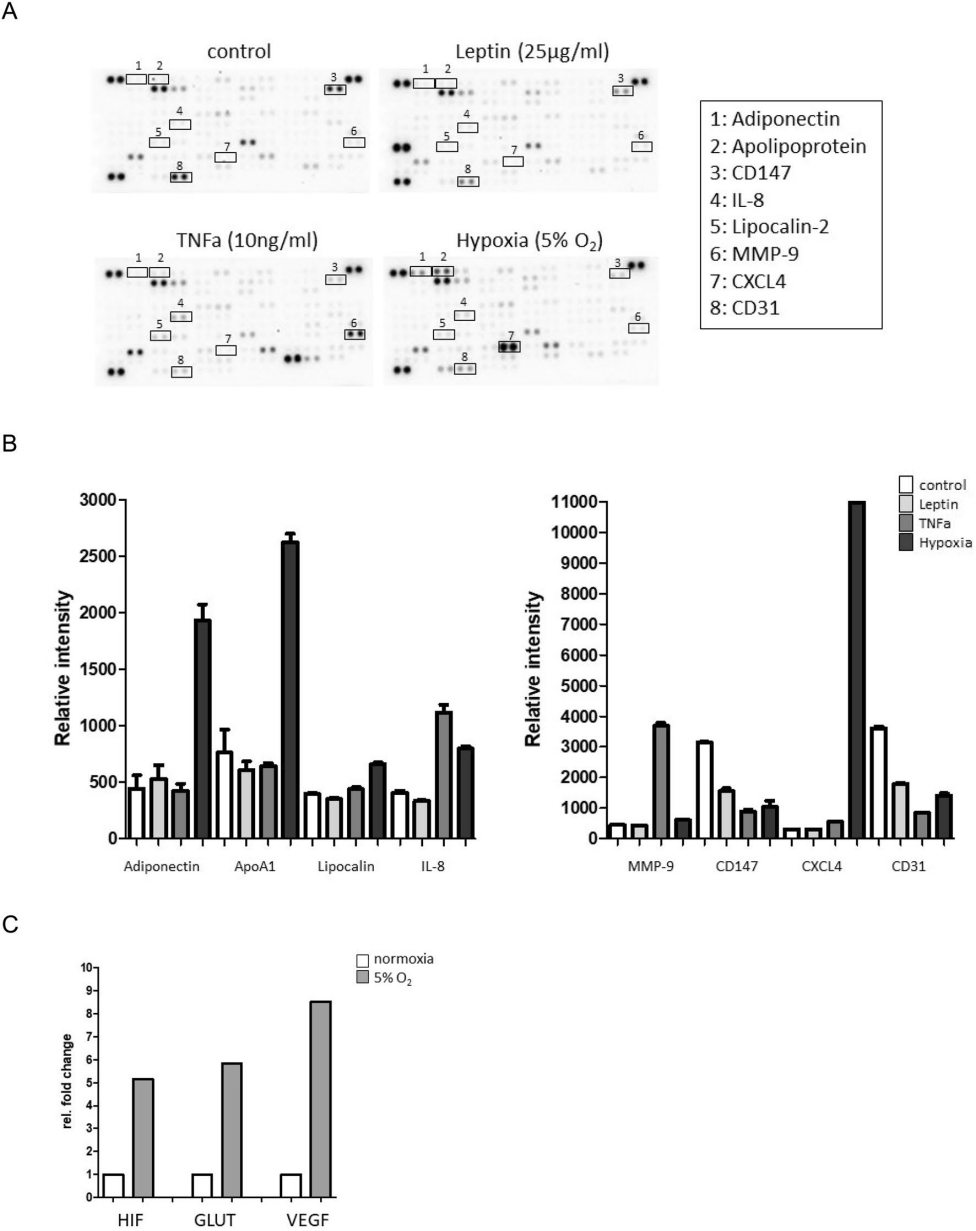
Incubation of THP-1 monocytes with OSAS and obesity related factors. (**A**) Raw images of cytokine arrays of THP-1 cell culture supernatants after 24 h of treatment with leptin (25 µg/ml), TNFα (10 ng/ml) and Hypoxia (5% O_2_) compared to the medium control. Numbers indicate differential densities of bands of certain cytokines (**1**: Adiponectin; 2: Apolipoprotein; 3: CD147; 4: IL-8; 5: Lipocalin; 6: MMP-9; 7: CXCL4; 8: CD31). (**B**) Semiquantitative analysis was performed by measuring the density of the dots and revealed differential secretion patterns of different cytokines (Adiponectin; Apolipoprotein; CD147; IL-8; Lipocalin; MMP-9; CXCL4; CD31) in response to leptin, TNFa and hypoxia treatment compared to the internal medium control. (**C**) Expression levels of hypoxia inducible genes VEGF, GLUT1 and HIF-1α via quantitative PCR after 24 h of incubation of THP-1 monocytes at 5% O_2_. Gene-specific mRNA expression was measured using the ΔΔCt method relative to expression of ribosomal protein L28 (endogenous control) and normalized to appropriate normoxic controls.

To analyze cytokine secretion of THP-1 monocytes in responses to hypoxia, tumor necrosis factor α (TNFα) and leptin, the expression patterns of 105 different cytokines and chemokines in supernatants of the treated monocyte cell cultures were determined using human cytokine antibody arrays (Fig. 5A).

Semiquantitative analyses were performed by measuring the density of the dots and revealed differential secretion patterns of different cytokines in response to the different incubation parameters compared to the internal medium control (Fig. 5B).

**Figure 6:**
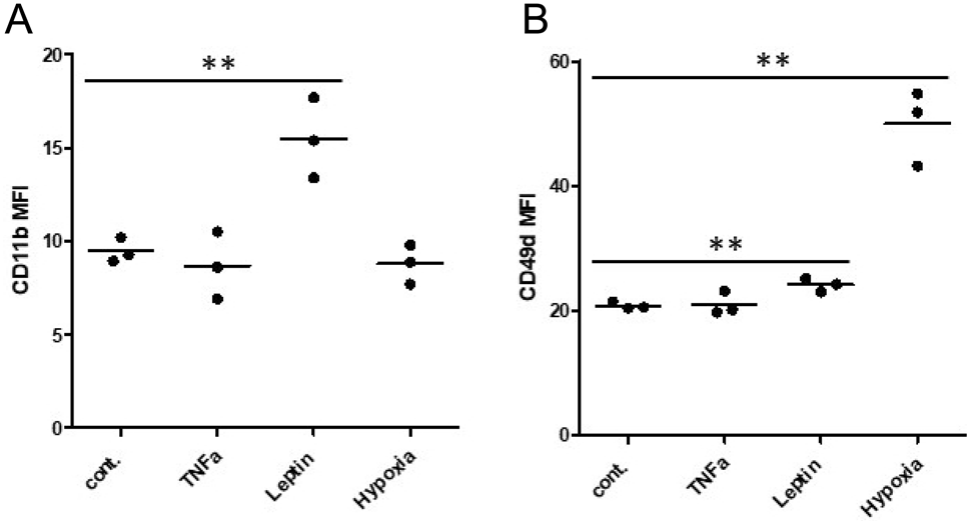
Adhesion molecules on THP-1 monocytes in response to leptin (25 µg/ml), TNFα (10 ng/ml) and Hypoxia (5% O_2_) compared to the internal medium control. Measurements (n = 3) revealed significantly increased expression levels of (**A**) CD11b in response to leptin and (**B**) CD49d in response to leptin and hypoxia. **: *p* < 0.01; MFI: mean fluorescence intensity.

Induction of intermittent hypoxia was confirmed by increased expression levels of hypoxia inducible genes VEGF, GLUT1 and HIF-1α via quantitative PCR after 24 h incubation of THP-1 monocytes at 5% O_2_ (Fig. 5C). Moreover, analyses of different adhesion molecules and chemokine receptors on incubated THP-1 monocytes revealed significantly increased expression levels of CD11b in response to leptin (*p* = 0.0097) and significantly increased expression levels of CD49d in response to 5% hypoxia (*p* = 0.0011) (Fig. 6).

## Discussion

### Alteration of circulating monocyte subsets

Aim of this study was to investigate the influence of OSAS, obesity and certain related factors on the abundances and characteristics of peripheral blood monocyte subsets and THP-1 monocytes.

In order to distinguish these overlapping diseases, whole blood analyses were performed from normal weight OSAS patients, patients with obesity but without OSAS, and patients with OSAS and obesity as compared to healthy donors. Analyses revealed highly significant redistributions of circulating classical monocytes and intermediate monocytes in all three patient cohorts, which underlines that both conditions favor a low-grade systemic inflammation and immune alterations^2,3,16^. These findings corroborate recent data where monocyte subset distributions of OSAS patients could be normalized by therapeutic hypoglossal nerve stimulation (HNS) and the corresponding positive effects on the peripheral blood oxygenation^18^. Adhesion of monocytes to endothelial cells is a crucial step in the initiation of atherosclerosis and cardiovascular disease, which are the main comorbidities of patients suffering from obesity and/or OSAS due to the underlying systemic inflammation^19^.

Analysis of monocytic adhesion molecules revealed elevated expression levels of CD11a and CX3CR1 in response to both disease conditions, whereas CD11b was elevated in patients with OSAS and patients with OSAS and obesity but not in the obesity cohort.

Expression levels of adhesion molecule CD11c were oppositely affected in the obesity cohort as compared to normal weight patients with OSAS and patients with OSAS and obesity and thus further disentangle recent data on monocytic adhesion molecules in obese patients with OSAS^16^. It has been shown in patients with obesity, that CD11c + innate immune cells in adipose tissue are involved in the formation of an inflammatory state and insulin resistance^20^. Integrins CD11a and CD11b are well known leukocyte adhesion molecules, that are associated with the adhesion of inflammatory monocytes to endothelial cells in patients with coronary artery diseases^21^. CX3CR1 is well known to be associated with atherosclerosis and vascular inflammation^22,23^. Adhesion molecule CX3CR1 is essential for monocyte crawling along the blood vessels and the interaction with endothelial cells^24^. CD16^+^ monocyte subsets in both human and mouse have been described to express high levels of CX3CL-1 (CX3C-chemokine ligand 1) receptor CX3CR1^24^. Increased expression levels of monocytic CX3CR1 in patients with obesity were also shown in an earlier study, but without distinction of the individual OSAS status^25^. These data corroborate the multifactorial impact of OSAS and obesity with a clear need to disentangle pathophysiological changes in both diseases. Of note, our data revealed significantly decreased expression levels of CD29 in all three monocyte subsets in patients with OSAS and obesity but not in the obesity or OSAS cohort. Adhesion molecule CD29 is an important mediator of the adhesiveness between leukocytes and endothelial cells^26^ and a reduced inflammation of bacterial colitis in mice has been demonstrated in response to blockade of CD29 using inhibitory antibodies^27^.

These data are the first step to distinguish the impact of both diseases, which occur together in the majority of patients. For instance, CD11b is increased in patients with an OSAS background, independently of the presence of obesity, whereas adhesion molecules CD29 was significantly decreased through the interaction of both diseases. In this context, it is important to mention, that the cohort of normal weight OSAS patients suffer from a rather mild OSAS compared to the cohort of patients with OSAS and obesity. However, further comprehensive investigations on larger patient cohorts are required to find out whether obesity and OSAS just share some common immunological effects and whether they are additive or synergistic.

### Plasma inflammatory mediators

Systemic inflammation in patients with OSAS or obesity is characterized by alterations in the circulating levels of several cytokines, which contribute to immune disturbances and the development of concomitant diseases^28–30^.

It is well known that adipose tissue is one of the main sources of pro-inflammatory cytokines, which are involved in the process of monocyte rolling, adhesion and extravasation and the development of atherosclerosis^31–33^. In this respect, significantly higher expression levels of pro-inflammatory cytokines and pro-inflammatory macrophages were found in visceral adipose tissue compared to subcutaneous adipose tissue^34–36^.

Our data revealed differential expression patterns of plasma adiponectin, leptin, lipocalin and MMP-9 in the analyzed patient cohorts. Significantly increased levels of plasma adiponectin have been found in normal weight patients with OSAS. So far, there were controversial data whether adiponectin acts as an anti- or pro-inflammatory factor^37–39^ and the association of blood levels of adiponectin in OSAS patients was a mostly unknown issue with conflicting results^40^.

Moreover, our data revealed significantly elevated plasma leptin levels in the obesity cohort and a significant positive correlation with the BMI values. These data corroborate earlier data with regard to obese patients with OSAS^16^. It is well known that leptin is secreted by white adipose tissue in direct relation to fat mass and involved in the regulation of cellular inflammatory processes^41–43^. Interestingly, inflammatory monocytes in patients with asthmatic disease have recently as well been identified as leptin-producing cells^44^. The impact of obesity and OSAS on plasma leptin levels is up to now controversially discussed in the literature. Some studies suggested that OSAS patients have higher plasma levels of leptin^45^, while others observed no differences in serum leptin between OSAS patients and controls^46^. Our data clearly underline that elevated fat mass as seen in obesity is the major amplifying factor among these two conditions^46^. Moreover, it has been shown that leptin also participates in the regulation of cell mediated inflammatory processes via increased numbers of granulocytes, NK cells, monocytes and hematopoietic progenitors^43,47^. Increased plasma lipocalin levels have been found in both normal weight patients with and patients with obesity but without OSAS. Lipocalin is an acute-phase protein and has recently been shown to play an important role in inflammation and oxidative stress response following acute lung injury^48^. In patients with obesity, an activation of macrophages has been shown to occur via adipocyte-derived lipocalin^49^. These data corroborate the positive correlation between plasma lipocalin levels and BMI values in patients with obesity in the present study. Furthermore, we measured significantly increased plasma MMP-9 levels in both the OSAS cohort and the obesity cohort, which positively correlated with BMI and AHI values. This is in line with recent data which revealed a positive correlation of serum MMP-9 with the severity of OSAS^50^ and increased levels of pro-inflammatory MMP-9 that has been shown in the saliva of patients with obesity with non-alcoholic fatty liver disease^51^.

### Impact on THP-1 monocytes

Our data revealed differential cytokine secretion patterns of THP-1 monocytes in response to leptin, TNFα and hypoxia, where particularly increased secretion levels of adiponectin, apolipoprotein (ApoA1) and chemokine CXCL4 by THP1 monocytes in response to 5% hypoxia stood out.

Apo1 has been suggested as promising novel biomarker for diagnosis of coronary artery disease^52^ and it has been shown that ApoA1 is associated with pathological angiogenesis in hypoxia-induced human retinal vascular endothelial cells by inhibiting ERK1/2 signaling^53^.

It has recently been shown that hypoxia is fundamental for CXCL4 production by umbilical cord CD34 derived plasmacytoid dendritic cells, via an overproduction of mitochondrial reactive oxygen species (mtROS) and the stabilization of HIF-2α^54^. TNFα induced elevated secretion levels of IL-8 and MMP-9, whereas leptin did not lead to increased cytokine secretion patterns. These data corroborate earlier studies, which have shown that TNFα up-regulates MMP-9 expression via NF-κB pathways within the regulation of bone inflammatory diseases^55^ and also contributes to the inducible expression of IL-8 in response to oxidative stress^56^.

In summary, our study revealed differential effects of OSAS and obesity and its related factors on the systemic balances of inflammatory mediators and immunological characteristics of human monocyte subsets. An acknowledged limitation of the study is the relatively small patient cohort. Further ongoing comprehensive investigations on larger and gender balanced patient cohorts, also in terms of different stages of disease (obesity class I-III; mild, moderate, severe OSAS), before and after therapeutic interventions are needed to better understand the complex regulation of the systemic immunity in these patients.

## Materials and methods

### Ethics statement and blood collection

All patients were clinically examined at the Department of Otorhinolaryngology and the Department of Internal Medicine 1, University Hospital Schleswig–Holstein, Campus Luebeck. The study was approved by the local ethics committee of the University of Luebeck (approval number 21-183) and conducted in accordance with the ethical principles for medical research in humans as stated by the WMA Declaration of Helsinki. All blood donors have signed an informed written consent, and were clarified about the aims of the study and the use of their samples. Blood (8 ml) was drawn by venipuncture into a sodium citrate containing S-Monovette (Sarstedt; Nümbrecht, Germany). According to WHO guidelines, individuals can be subdivided based on their BMI (body mass index; kg/m^2^) values into overweight (BMI 26–30), obesity class I, (BMI 31–35) obesity class II (BMI 36–40) and obesity class III (BMI above 40). In our study, blood samples were collected from healthy donors (n = 10, 5 female/5 male; mean age of 36.4, body mass index (BMI) of 24.7 kg/m^2^), normal weight OSAS patients with normal BMI (n = 10, 3 female/7 male; mean age of 48.7, BMI of 25.7 kg/m^2^), obese patients without OSAS (n = 10, 7 female/3 male; mean age of 39.8, BMI of 39.8), and obese patients with OSAS and obesity (n = 10, 7 female/3 male; mean age of 35.7, BMI of 43.7). All included obese patients in our study revealed BMI values higher 35. Moreover, different clinical parameters of obese patients were diagnosed such as diabetes mellitus, non-alcoholic fatty liver disease, hypertension, lipid-lowering medications, cholesterol values (LDL in mmol/l), and glycated hemoglobin (HbA1c in %) values, respectively. The clinicopathological characteristics of obese patients are listed in Table 1.

**Table 1:** Clinicopathological parameters of obese patients without OSAS.

Characteristics	Obese patients without OSAS (n = 10)	
	n	%
Diabetes		
Yes	2	20
No	8	80
Fatty liver		
Yes	10	100
No	0	0
Hypertension		
Yes	4	40
No	6	60
Lipid lowerig		
Yes	1	10
No	9	90
Cholesterol (mmol/l)		2.8 ± 0.58
HbA1c (in %)		5.4 ± 0.77
AHI		3.9 ± 0.6
ODI		1.3 ± 1.5

Different clinical parameters of obese patients were diagnosed such as diabetes mellitus, non-alcoholic fatty liver disease, hypertension, lipid-lowering medications, cholesterol values (LDL in mmol/l), and glycated hemoglobin (HbA1c in %) values, respectively. Values are presented as mean ± SD.

All included OSAS patients in our study revealed an AHI (apnea–hypopnea index) values higher 15 events per hour in polysomnography (REMbrandt™ Version 9.1, Natus Medical Incorporated, USA) or an AHI higher 5 events per hour with additional OSAS comorbidities such as hypertension and/or with excessive daytime sleepiness. Apneas were defined as a complete breathing cessation for at least 10 s and hypopneas as breathing restriction by 30% accompanied by a drop of oxygen saturation of at least 4%^57^. Patients can be subdivided into mild OSAS (AHI 5–14/h), moderate OSAS (15–30/h), and severe OSAS (≥ 30/h). In addition, ODI (oxygen-desaturation-index) values were measured, which refers to the average number of desaturation episodes occurring per hour, where desaturation episodes are defined as a decrease in the mean oxygen saturation of ≥ 3% that lasts for at least 10 s^58^. Oxygen levels are considered abnormal when they drop below 90%. Therefore, the percentage of cumulative time with oxygen saturation below 90% in total sleep time (T90) was measured. Moreover, excessive daytime sleepiness of OSAS patients was evaluated using the established Epworth Sleepiness Scale (ESS) questionnaire^59^, which measures the probability of falling asleep in a variety of everyday situations and indicates underlying sleep disorders or medical conditions. For the German version, ESS scores ≥ 11 points indicate excessive daytime sleepiness^60^. Overall, our cohort normal weight OSAS patients suffering from a mild OSAS, most likely because of not being overweight. The clinicopathological characteristics of normal weight OSAS patients are listed in Table 2.

**Table 2:** The clinicopathological characteristics of OSAS patients.

Characteristics	Normal weight OSAS patients (n = 10)
AHI (events/h)	12.4 ± 3.9
ODI (events/h)	1.0 ± 0.7
ESS	9.9 ± 4.5
O_2_ saturation (T90)	1.5 ± 2.5

Clinical parameters of OSAS patients as median AHI (apnea–hypopnea index), ODI (oxygen desaturation index), ESS (Epworth Sleepiness Scale) and O_2_ saturation (T90). Values are presented as mean ± SD.

Distribution and expression of adhesion molecules from normal weight OSAS patients and patients with obesity but without OSAS were compared with patients with OSAS and obesity, which have already been analyzed in an earlier publication^16^. The clinicopathological characteristics of patients with OSAS and obesity are listed in Table 3.

**Table 3:** Clinicopathological parameters of patients with OSAS and obesity.

Characteristics	Patients with OSAS and obesity (n = 10)	
	n	%
Diabetes		
Yes	3	30
No	7	70
Fatty liver		
Yes	10	100
No	0	0
Hypertension		
Yes	6	60
No	4	40
Lipid lowerig		
Yes	4	40
No	6	60
Cholesterol (mmol/l)		2.4 ± 0.94
HbA1c (in %)		6.5 ± 1.90
AHI		23.5 ± 9.1
ODI		19.2 ± 6.6

Different clinical parameters of obese patients were diagnosed such as diabetes mellitus, non-alcoholic fatty liver disease, hypertension, lipid-lowering medications, cholesterol values (LDL in mmol/l), and glycated hemoglobin (HbA1c in %) values, respectively. Values are presented as mean ± SD.

### THP-1 cells and culture conditions

For cell culture experiments the non-adherent monocyte cell line THP-1 (Tohoku Hospital Pediatrics-1)^17^ was used. Cell culture was performed in RPMI 1640 medium supplemented with 10% heat inactivated fetal bovine serum (FBS), 1% sodium pyruvate and 1% streptomycin/penicillin at 37 °C and 5% CO_2_ under a humidified atmosphere. Cells were subcultured every 3 days when they reached a maximum density of 1 × 10^6^ cells/ml. For condition of intermittent hypoxia (5% O_2_), cells were incubated in a humidified incubator (Heracell Vios 160i Co2-Incubator, Thermo scientific, Waltham, MA, USA) at 37 °C for 24 h. Further, THP-1 cells were incubated with Leptin (25 µg/ml) and tumor necrosis factor α (TNFα) (10 ng/ml) (Bio-Techne GmbH, Wiesbaden, Germany) for 24 h, respectively.

### Inflammatory mediators

The levels of plasma inflammatory mediators adiponectin, leptin, lipocalin and metalloproteinase 9 (MMP-9) were determined from EDTA-plasma samples using enzyme-linked immunosorbent assays (ELISA) according to the protocols given by the commercial ELISA kits (R&D Systems, Minneapolis, MN, USA). Comprehensive analysis of THP-1 cytokine expression patterns in responses to leptin (25 µg/ml), tumor necrosis factor α (TNFα) (10 ng/ml) and intermittent hypoxia (5% O_2_) was performed using human cytokine arrays. Therefore, supernatants from cell cultures were collected after incubation and instantly frozen with liquid nitrogen and preserved at − 80 °C. Proteome Profiler™ Human XL cytokine array (R&D Systems, Minneapolis, MN, USA) was hybridized with the cell culture medium as recommended by the supplier.

### Staining of THP-1 cells and FACS analysis

For primary monocytes, 20 µl of citrate blood was diluted in 80 µl PBS for staining within 4 h after blood collection. Primary monocytes and THP-1 cells were stained with following antibodies (diluted 1:50): CD45-PE (Cat: 368510), CD14-FITC (Cat: 367116), CD16-BV-510 (Cat: 302048), HLA-DR-APC-Cy7 (Cat: 307618), CX3CR1-BV421 (Cat: 341620), CD11a-PE-Cy7 (Cat: 301220), CD11b-PerCP (Cat: 101230), CD11c-BV421 (Cat: 371512), CD29-PE-CY7 (Cat: 303026), and CD49d-APC (Cat: 304308) (all from Biolegend, San Diego, USA). After 25 min staining in the dark, 650 µl RBC Lysis Buffer (Biolegend) were added to the samples and incubated for another 20 min. Subsequently, suspension was centrifuged at 400 × g for 5 min and supernatant was discarded. Cell pellet was resuspended in 100 µl fresh PBS and used for FACS analysis (Supplementary Figure 1S). Flow cytometry was performed with a MACSQuant 10 flow cytometer (Miltenyi Biotec, Bergisch-Gladbach, Germany) and data were analyzed using the FlowJo software version 10.0 (FlowJo, LLC, Ashland, USA).

### Total RNA extraction and quantitative real-time PCR

Total RNA from primary and THP-1 monocytes was extracted using the innuPREP RNA Mini Kit 2.0 (IST Innuscreen GmbH, Berlin, Germany) according to the manufacturer’s protocol. cDNA of total RNA (100–300 ng) was synthesized with the M-MuLV reverse transcriptase (New England Biolabs, Frankfurt, Germany) and random hexamer primers (ThermoFisher Scientific) according to the instructions of the manufacturer. The sequences of forward and backward primers are listed in Table 4.

**Table 4:** Sequences of forward and backward primers.

Primers	Sequences
VEGF	
Forward	5′- CGAGGCAGCTTGAGTTAAACG -3′
Reverse	5′- AGATCTGGTTCCCGAAACCCT -3′
GLUT1	
Forward	5′- GGCCTTTTCGTTAACCGCTT -3′
Reverse	5′- AGCATCTCAAAGGACTTGCCC -3′
HIF-1α	
Forward	5′- ACACACAGAAATGGCCTTGTGA-3′
Reverse	5′- GTGAATGTGGCCTGTGCAGT -3′
L28	
Forward	5′- ATGGTCGTGCGGAACTGCT -3′
Reverse	5′- TTGTAGCGGAAGGAATTGCG -3′

All primers were synthesized by Invitrogen (USA). Quantitative RT-PCR was performed in the Eco48 qPCR System (PCRmax Limited Beacon Road, Staffordshire, United Kingdom) using 1 μL cDNA and the SensiMix SYBR Kit (Bioline, Luckenwalde, Germany) in a total volume of 12.5 μL per assay. The cutoff point (Ct) was defined as the value when the fluorescent signal increased above the background threshold. Gene-specific mRNA expression of hypoxia inducible genes (VEGF, GLUT1, HIF-1α) was normalized to mRNA expression of ribosomal protein L28 (RPL28). Relative expression values were calculated using the 2^∆∆ct^-method and are presented as the fold induction to L28 compared with THP-1 monocytes cultured in normoxia.

### Statistical analyses

Statistical analyses were performed with GraphPad Prism Version 7.0f. The mean and standard error (SEM) are presented. The differences between groups were determined after testing for Gaussian distribution (normality tests), and applying parametric (student`s t-Test), or non-parametric 1-way ANOVA with Bonferroni post hoc test. Power calculations for sample size were performed to ensure statistical power of 0.8 or higher and revealed an associated required sample size of at least n = 6 per cohort. The correlation between parameters was calculated using multivariate regression with the Pearson correlation coefficient. p < 0.05 (*), p < 0.01 (**), and p < 0.001 (***). Additional statistical details such as sample size are given in the respective figure legends, when appropriate.

### Supplementary Information

Supplementary Figure S1.

## Data Availability

The datasets used in the current study are available from the corresponding author on reasonable request.
