# Supplementary material for: Distinguishing the impact of distinct obstructive sleep apnea syndrome (OSAS) and obesity related factors on human monocyte subsets
Source: Sci Rep. 2024 Jan 3;14:340. doi: 10.1038/s41598-023-49921-5 (PMC10764945; doi:10.1038/s41598-023-49921-5)
Supplement: Supplementary file 1 — Supplementary Figure S1. [file 41598_2023_49921_MOESM1_ESM.docx]

**Supplementary material**


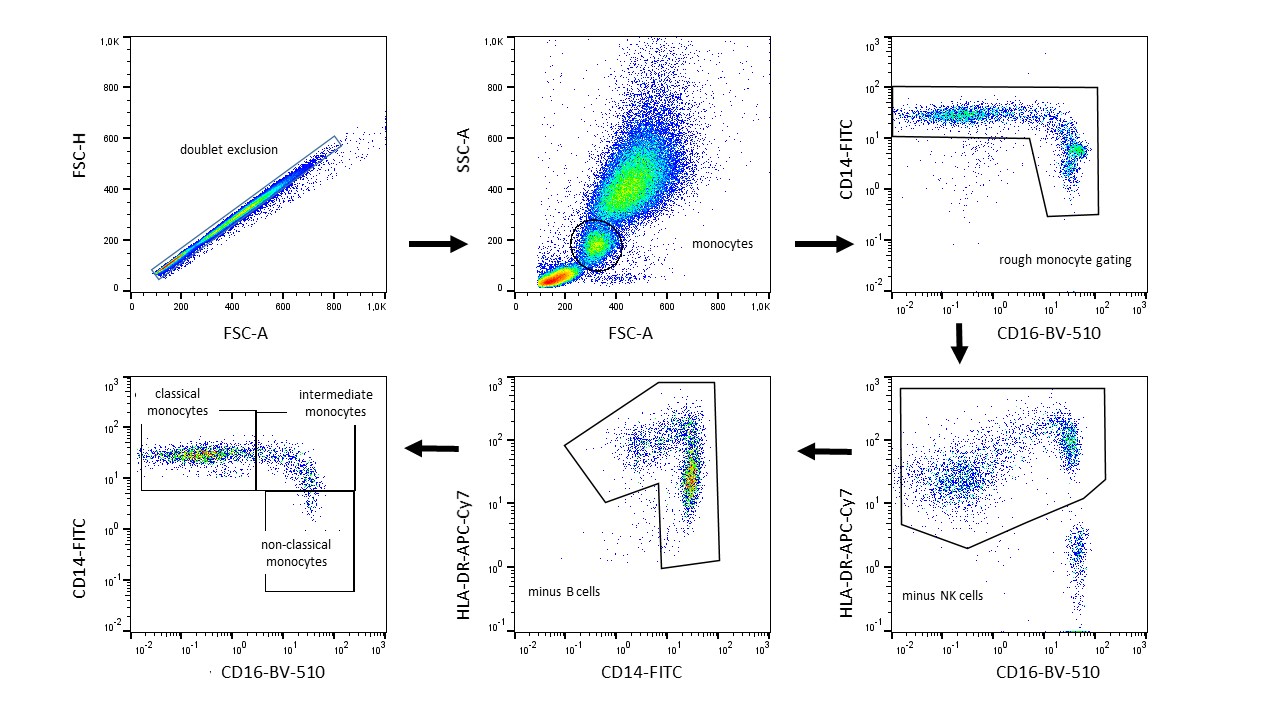


**Figure 1S:** Representative example gating for flow cytoemtric analysis of monocyte subsets.

After doublet exclusion of CD45+ leucocytes, a rough gating of monocytes was first carried out by their forward scatter (FSC) and side scatter (SSC) characteristics and further by their CD14 and CD16 expression. NK cells and neutrophil granulocytes were excluded by their missing HLA-DR expression and remaining B cells by their missing CD14 expression. Finally, monocyte subsets were subdivided into CD14^++^CD16^-^ “classical” monocytes, CD14^+^CD16^+^ “intermediate” and CD14^dim+^CD16^+^ “non-classical” monocytes.
